# Bioactive Components and Health Benefits of Saskatoon Berry

**DOI:** 10.1155/2020/3901636

**Published:** 2020-05-14

**Authors:** Lunan Zhao, Fei Huang, Amy Leung Hui, Garry X. Shen

**Affiliations:** ^1^Mansfield College, University of Oxford, Oxford OX1 3TF, UK; ^2^Department of Internal Medicine, University of Manitoba, Winnipeg, Manitoba, Canada R3E 3P4

## Abstract

In response to the recent rise in numbers of diabetes patients, many treatments have been developed; but currently, oral antihyperglycemic agents and insulin are still the main clinical treatments. Since current drugs have limitations and harmful side effects, research in alternative treatments has been sought. This article reviews recent research updates of Saskatoon berries (SB), covering its background information, its main active ingredients, its structure, and its function. Flavonoid compounds in Saskatoon berries, in particular flavanol, anthocyanin, and proanthocyanin, possess anti-inflammatory, antitumor, and antidiabetes impacts. The current review synthesizes the latest research on the health benefits of Saskatoon berry in a variety of domains. With further research, SB has the potential to help treat and prevent diabetes in the future.

## 1. Introduction

Saskatoon berry plant (*Amelanchier alnifolia Nutt.*, also referred to as Saskatoon, chuckley pear, juneberry, western juneberry, serviceberry, pacific serviceberry, western serviceberry, alder-leaf shadbush, dwarf shadbush, prairie berry, and pigeon berry), is a type of tall shrub naturally growing in western regions of North America, including the southern regions of the Yukon and Northwest Territories, Canadian prairies (Alberta, Saskatchewan, and Manitoba), and northwestern states of the United States. The plant grows in a variety of soil conditions and survives at -60°C temperature. It features white flowers in the spring and produces red to purple berries in the summer. Indigenous people in North America harvest Saskatoon berry and mix it with raw buffalo meat and animal fat to make Pemmican, which is a type of preserved food. Dried Pemmican was used as survival food for Native hunters during long trips (Amy S 2016). The ripen Saskatoon berry may be stored freeze-dried to ensure availability throughout the year [1–5].

Although the Saskatoon berry appears similar to the blueberry, they are more closely related to the apple family and belong to the family Rosacea. Saskatoon berries have an excellent sweet, nutty almond flavor and appear on the market as fresh fruit or processed jam. They are important economic produce in Canada, being the second most planted fruit in Saskatoon, following strawberries [1, 4, 6]. Historically, they were applied for medicinal purposes. The early settlers in the North American prairies used the Saskatoon berry to disinfect and to prevent miscarriages, in addition to a source of food [7]. The Blackfoot Indigenous group made a tea from serviceberry twigs and leaves to treat diabetes [8, 9]. In North America today, its medicinal properties remain underexplored and it is consumed primarily as syrups, pies, jams, spreads, jellies, bakery goods, cereal, wines, cider, beers, snack food, and ice cream [4, 7, 10]. The primary cultivars of Saskatoon berry include Bluff, Elizabeth (specifically in Saskatoon), Martin, Honeywood, Lee, Martin, Nelson, Northline, Parkhill, Pearson II, Pembina, Regent, Smoky, Success, Thiessen, and Tisnovsky. Popular cultivars are Honeywood, Northline, Martin, Smoky, Pembina, and Thiessen [2, 4, 6, 11].

Since the mid-1960s, commercial orchards started to grow Saskatoon berry plants in Canada. In the last two decades, Saskatoon berry agriculture was expanded from North America to other European countries, including Poland, Finland, and the Czech Republic [10, 12, 13]. The increasing popularity of the Saskatoon berry is often attributed to its nutritional values, such as supplemental sources of polyphenols, fiber, elements, vitamins, and antioxidants [3, 6, 11, 14]. Saskatoon berries contain high quantities of manganese, iron, potassium, vitamins (ascorbic acid, folic acid, pantothenic acid, pyridoxine, riboflavin, thiamin, and tocopherol), pectin, and carotene. In addition to minerals, Saskatoon berries possess phenolic acids, including 3-feruloylquinic, chlorogenic, and 5-feruloylquinic acids [3, 6, 13, 14]. Saskatoon berries have high quantities of anthocyanins (mainly cyanidins) and other types of flavonoids, such as rutin, hyperoside, avicularin, and quercetin [2, 3, 6, 11, 14].

## 2. Structure and Functions of Flavonoids

**Figure 1:**
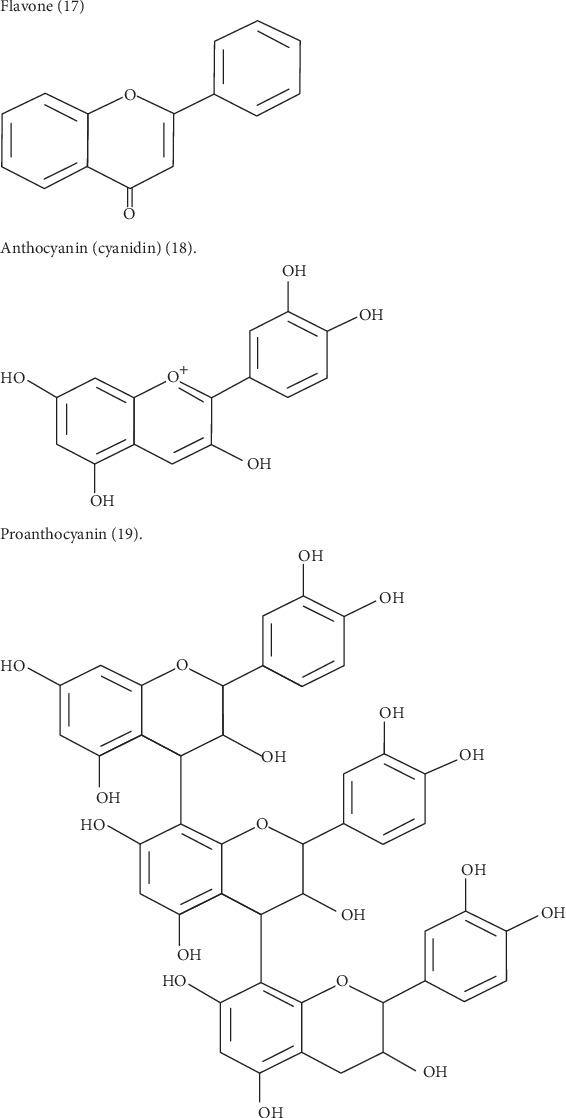
Three major subclasses of flavonoids. Flavone [17]. Anthocyanin (Cyanidin) [18]. Proanthocyanin [19].

Saskatoon berries have a high content of polyphenols [15], which are possibly the major functional components in Saskatoon berries [6, 11]. The primary phenolic compounds in Saskatoon berries are flavonoids. Flavonoids encompass over 6000 phenolic substances that are found in pigmented fruits, leaves, vegetables, grain seeds, and their products, such as tea, soy, jam, juice, and wine [6, 11]. The high content of flavonoids in the fruit is responsible for its observed anti-inflammatory, antidiabetic, and chemo-protective effects [4]. Structurally, flavonoids consist of two aromatic rings (A and B rings) linked by a 3-carbon chain that forms an oxygenated heterocyclic ring (C ring). The seven subclasses of flavonoids include flavanes, flavanols (proanthocyanidins), flavanones, flavones, isoflavones, flavonols, and anthocyanidins. The distinction between subclasses results from different generic structures of the C ring, functional groups on the rings, and positions at which the B ring is attached to the C ring. Within each subclass, individual compounds are characterized by specific hydroxylation and conjugation patterns [16], (Figure 1).

**Figure 2:**
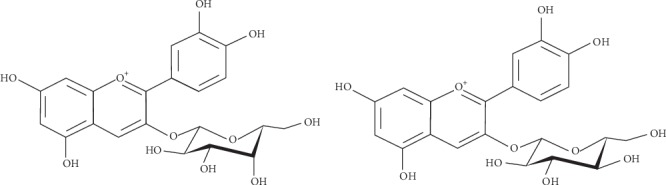
Major anthocyanins found in Saskatoon Berry [23].

Among the subclasses of flavonoids, anthocyanidins in a glycan form (anthocyanins) are the largest group of water-soluble natural pigments in the plant kingdom. They are responsible for most of the red, violet, and blue colors of fruits, vegetables, leaves, and seeds. Anthocyanins are strong antioxidants [20]. Other anthocyanidins include delphinidin, malvidin, peonidin, and petunidin. Cyanidin and pelargonidin color plants red and purple, whereas delphinidin is responsible for bluish colors in plants. In plants, anthocyanins occur in a 3- or 3,5-glycosylated form of anthocyanidins (aglycones) and are generally linked with glucose, galactose, arabinose, rhamnose, xylose, or fructose. In fruits, the primary anthocyanins are glycosides composed of six anthocyanidins [21]. Cyanidin-3-galactoside and cyanidin-glucoside are the most abundant anthocyanins in Saskatoon berry [3, 19, 27], (Figure 2).

Another major class of flavonoids is proanthocyanidins (flavans, condensed tannins), which have a brown oxidized color and provide flavor and astringency to fruits [14]. The classification of proanthocyanidins depends on the degree of polymerization, which indicates the number of linked monomers, the hydroxylation patterns of basic units, and the links between units. The individual oligomers are commonly referred to as dimers, trimers, tetramers, pentamers, hexamers, and heptamers [24]. Flavan-3-ol units compose proanthocyanidins, which is found widely in many types of fruits, nuts, seeds, and bark of pine [10]. The primary constitutive units are epicatechins and epigallocatechins, which form procyanidin and prodelphinidin structures, respectively [10, 24]. Procyanidins represent the largest class of proanthocyanidins [18]. Flavan-3-ol units often connect through B-type bonds, such as C4 ⟶ C8, and to a lesser extent, C4 ⟶ C6 linkages. Albeit less common, the flavan-3-ol units can also be linked by an additional C2 ⟶ O7 or C2 ⟶ O5 linkage (doubly bonded A-type). Proanthocyanidins are powerful antioxidants, and they have also exhibited other potential health benefits such as anticarcinogenic, anti-inflammatory, and vasodilatory activities. Their physical, chemical, and biological characteristics depend largely on their structures and particularly on their degree of polymerization [10, 24].

**Figure 3:**
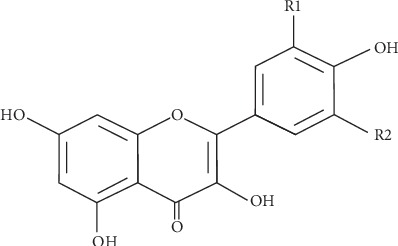
Flavonol, a subclass of flavonoids [25].

A third common class of flavonoids is flavonols, which have a 3-hydroxyflavone backbone (Figure 3). Their diversity stems from the different positions of the phenolic-OH groups. Flavonols are found in an assortment of substances, such as tea, lovage, pepper, coriander, fennel, radish, dill, berries, onions, apples, and wine. One of the common types of flavonols in human diets is quercetins, which is the major flavonol aglycone. Other types of flavonols include rutin and quercitrin [4, 16]. Most of those flavonoids have antioxidant activities.

## 3. Bioactive Components in Saskatoon Berry

Saskatoon berries contain abundant amounts of minerals, such as calcium, copper, iron, magnesium, manganese, and potassium, as well as vitamins (ascorbic acid, folic acid, pantothenic acid, pyridoxine, riboflavin, thiamin, and tocopherols) and fiber [3, 6, 13, 14]. Besides micronutrients, mature Saskatoon berries contain significant levels of the anthocyanins, flavonol, and proanthocyanidin classes of flavonoids [3, 14, 21]. The major type of anthocyanins in Saskatoon berries are cyanidin-based anthocyanins, which comprised 63% of all phenols and 94% of anthocyanins in Saskatoon berry [6, 19]. The content of anthocyanins is often low in young fruits and dramatically increases from stage 5 to 9 of the nine-stage maturation of fruits [4, 14]. The abundances of anthocyanin concentrations in different Saskatoon berry cultivars varied [3]. Hosseinian and Beta reported that ripen Smoky Saskatoon berry has a comparable level of total anthocyanins as that in wild blueberry and a higher level than that in raspberry, sea buckthorn, chokeberry, or strawberry [21]. Cyanidin 3-O-galactoside (C3Ga), cyanidin 3-O-glucoside (C3G), cyanidin 3-O-arabinoside, and cyanidin 3-O-xyloside are the most abundant anthocyanins in ripe Saskatoon berry [3, 11, 18, 19, 26, 27]. In Saskatoon berry, the content of C3G in Saskatoon berry (117.67 mg/100 g) is significantly higher than wild blueberry (27.48 mg/100 g), raspberry (35.88 mg/100 g), strawberry (9.53 mg/100 g), chokecherry (46.01 mg/100 g), and sea buckthorn (0.05 mg/100 g) [18]. The primary flavonols found in Saskatoon berries include the quercetin diglycosides (quercetin 3-O-rutinoside, quercetin 3-O-robinobioside, and quercetin 3-O-arabinoglucoside) and the quercetin monoglycosides (quercetin 3-O-galactoside, quercetin 3-O-glucoside, quercetin 3-O-arabinoside, and quercetin 3-O-xyloside) [11].

Not only do the fruits of the Saskatoon berry plant contain high contents of multiple phenolic compounds, such as anthocyanins, quercetin, and proanthocyanidin, but also the leaves, stems, and roots of the plant also contain high levels of phenolic compounds. The composition of phenolic compounds in other parts of the Saskatoon berry plant is different from that in fruits. For example, the main phenolic components in the leaves of the Saskatoon berry were quercetin 3-galactoside, chlorogenic acid, and (−)-epicatechin; the main compounds in the stem included flavonol and flavanone glycosides, catechins, and hydroxybenzoic acids [6, 7]. Limited studies have been done on the biological activity and health benefits of the nutrients and components of Saskatoon berry or other parts of the plant.

## 4. Biological Activity and Health-Promoting Effects of Saskatoon Berry and Its Active Components

Studies have demonstrated that anthocyanins, proanthocyanidins, flavonols, phenolic acids, and other phenolics have many protective functions, including playing antioxidant, antiradical, and potentially anticarcinogenic, anti-inflammatory, antidiabetic, vessel-protecting, and neuroprotective roles. They have potential preventative and therapeutic effects on many diseases such as cancers, inflammation and cardiovascular diseases, obesity, neurodegenerative pathologies, and muscular degeneration [6, 11, 14]. Because Saskatoon berries have high levels of beneficial flavonoids, such as anthocyanin, flavonol, and proanthocyanidin [3, 14, 21], research into its antioxidant, anti-inflammatory, antitumor, and antidiabetic properties have become a hot spot in recent years. Studies found that the total anthocyanin content significantly correlated to its antioxidant properties. The research results have suggested that three cultivars of Saskatoon berries, “Thiessen”, “Nelson”, and “Smoky”, exhibited high radical scavenging activity [4].

### 4.1. Antioxidant Effects

Because Saskatoon berries have strong activities of free-radical scavenging, they have antioxidant properties [10]. A study found that concentrated crude extract of Saskatoon berries inhibited nitric oxide production in activated macrophages, showing a potential protective role against chronic inflammation [7]. Cyclooxygenase (COX, including COX-1 and COX-2) is an enzyme that helps form prostanoids, such as thromboxane, prostaglandins, and prostacyclins, from arachidonic acid. Inhibiting COX with drugs can alleviate symptoms such as inflammation, blood clotting, and pain. Previous studies found that anthocyanin (100 ppm) mixtures inhibit COX-1 and COX-2 [4]. The findings suggest that the polyphenolic compounds contained in extracts of Saskatoon berries can effectively protect biological membranes, increase the resistance of low-density lipoprotein (LDL) to oxidation, influence lipid metabolism, and inhibit DNA damage [13, 15].

### 4.2. Anti-Inflammatory Effects

Studies have suggested that anthocyanins also attenuate the development of atherosclerotic cardiovascular diseases. The most common forms of anthocyanin in Saskatoon berries, as well as other field berries, are cyanidin-3-glucoside (C3G) and cyanidin-3-galactoside (C3Ga) [21, 27]. Another common type of anthocyanin is delphinidin-3-glucoside (D3G), the content of which is higher in Saskatoon berries than that in wild blueberry, raspberry, strawberry, chokecherry, and sea buckthorns in Manitoba [21]. These three compounds interfere at multiple points in the progression of cardiovascular disease. One classic risk factor for the disease is increased low-density lipoprotein (LDL). Studies found significantly high levels of glycated LDL (glyLDL) and oxidized LDL (oLDL) in type 1 and type 2 diabetic patients and coronary artery disease patients. Previous studies revealed the underlying mechanism of LDL's role, illuminating that glyLDL and oLDL elevated the level of reactive oxygen species (ROS), activated NADPH oxidase (NOX), and reduced the activities of mitochondrial electron transport chain (mETC) enzymes in vascular endothelial cells (EC). Subsequent studies found that the anthocyanins C3G, C3Ga, and D3G prevented glyLDL- and oLDL-induced upregulation of NOX activation, increased intracellular production of superoxides, increased endoplasmic reticulum (ER) stress, elevated unfolded protein response (UPR) markers and impairment of mETC enzymes, and decreased cell viability in cultured vascular EC. One important factor in glyLDL-induced oxidative stress in vascular EC is the blocking antibody for the receptor of advanced glycation end products (RAGE). Studies found that C3G decreased glyLDL-induced RAGE expression during the presence of RAGE antibody [21, 27, 28]. Shen et al. reported that C3G also has protective effects against the streptococcus suis serotype 2- (SS2-) mediated inflammatory response in J774 cell. C3G significantly suppressed the production of TNF-*α*, IL-1*β*, and IL-6 in cell supernatants. C3G also significantly inhibited multiple SS2-induced pathways, including inhibiting the phosphorylation of p38 to prevent the initiation of the mitogen-activated protein kinase (MAPK) and NF-*κ*B signaling pathways, as well as inhibiting Jun-N terminal kinase (JNK1/2), extracellular signal-regulated kinase (ERK1/2), and inhibitor *κ*B-*α* and NF-*κ*B p65 in J774 cells [29].

### 4.3. Anti-Tumor Effects

Many studies have also suggested that C3G may help with reproductive pathway issues and reducing tumors. Li et al. reported that C3G can be protective against cadmium- (Cd-) induced male reproductive dysfunction through regulating the hypothalamus-pituitary-gonadal (HPG) axis in male mice during puberty. C3G increased Gnrh1 gene expression in the hypothalamus, reversed the levels of gonadotropins (such as luteinizing hormone (LH) and follicle-stimulating hormone (FSH)), and improved the expression of LH and FSH receptor in the testis in mice exposed [30]. A key mechanism underlying tumor progression is angiogenesis. Studies found that C3G inhibited breast cancer-induced angiogenesis via attenuating the signal transducer and activator of the transcription 3 (STAT3)/VEGF pathway [31]. Harada et al. also evaluated the role of D3G on lipid accumulation induced by senescence in HepG2 (a human cell line derived from hepatocyte carcinoma). They found that D3G inhibited palmitic acid- (PA-) induced lipid accumulation and cellular senescence and reversed the expression of SMARCD1 to the level of the untreated hepatocytes [32]. Future studies will assess the variety of roles that anthocyanins play in biological pathways.

### 4.4. Impact on the Gut Microbiome

The gut microbiome plays an important role in the development of obesity, diabetes, and chronic inflammation. Limited studies have been conducted on the impact of Saskatoon berry on the gut microbiome. Zhao et al. investigated the effects of Saskatoon berry powder on gut microbiota in male C57BL/6J mice fed the high-fat high-sucrose diet for 15 weeks. The results indicated that the high-fat high-sucrose diet significantly increased the ratio of *Firmicutes/Bacteroidetes* at the phylum level compared with the control diet group. The ratio of *Firmicutes/Bacteroidetes* in feces of mice receiving high-fat high-sucrose diet supplemented with 5% Saskatoon berry powder was significantly lower than that in mice fed with high-fat high-sucrose. The reason for this ratio increase was because high-fat high-sucrose diets significantly decreased the abundance of *S24–7*, the major family bacteria in *Bacteroidetes* or “good” bacteria in the feces of mice. Supplementation of 5% Saskatoon berry resulted in a 40% increase in the abundance of *S24-7* family bacteria in the gut, but the change was not significantly different from high-fat high-ssucrose diets. The abundance of *S24–7* family bacteria was negatively correlated with glucose, insulin, HOMA-IR, body weight, cholesterol, triglyceride, PAI-1, TNF*α*, and MCP-1 [33]. The findings suggest that the administration of 5% Saskatoon berry powder improves the profile of gut microbiota by a relative increase in the abundances of good bacteria, which possibly contributes to its hypoglycemic, hypolipidemic and antiflammatic effects in mice.

### 4.5. Antidiabetic Effects of Saskatoon Berry

At present, the most common treatment for diabetes is still oral hypoglycemic drugs and insulin therapy. Traditional methods have played an extremely important role in the treatment of diabetes, yet they also have drawbacks. Alternative treatments to diabetes need to be explored and studied. One recent research area is the prevention and treatment of diabetes with natural substances. Bioactive components contained in Saskatoon berries, especially polyphenols, can reduce the blood glucose levels and regulate glycogen accumulation. For this reason, they can be useful in the treatment and prevention of diabetes [15]. Historical records and new research results in recent years have suggested that Saskatoon berries, as natural food, may be one of the best alternatives for the current medications used for preventing and treating diabetes.

In North America, indigenous people have used plants to treat diseases such as inflammation, cancer, and diabetes as part of their traditional practices. One example is the use of Saskatoon berries to treat diabetes. Johnston reported that one of the Indian tribes in North America, the Blackfoot Tribe, used the tea made from the leaves and twigs of Saskatoon berries to prevent and treat diabetes [9]. However, current research on the impact of Saskatoon berries on diabetes treatment is still rare. One molecule closely related to diabetic complications, such as cataract, neuropathy, kidney disease, retinopathy, and atherosclerosis, is aldose reductase. Drugs that block or attenuate aldose reductase activity can be used to prevent or delay the onset of diabetic complications. Plant-derived aldose reductase inhibitors are mainly classified into flavonoids, phenols and their derivatives, terpenoids, and alkaloids. In a study looking at the antidiabetic activity of Saskatoon berries, which contain a variety of flavonoids, Kraft et al. found that the nonpolar fraction of Saskatoon berries strongly inhibited aldose reductase (82% inhibition). The polar fraction of Saskatoon berries (including phenolic acids, anthocyanins, and proanthocyanidins) lowered blood sugar and strongly reduced the expression of IL-1*β* and COX-2. They also found that the berry samples can regulate lipid metabolism and energy expenditure, thereby ameliorating metabolic syndrome [7]. Other studies looked at the role of Saskatoon berries in inhibiting glucosidase, whose main function is to hydrolyze glycosidic bonds and release glucose into the blood. The enzyme is crucial in the sugar metabolism pathway of organisms, and it is divided into a *α*-type and a *β*-type. *α*-Glucosidase is directly involved in the metabolic pathway of starch and glycogen, and *β*-glucosidase is mainly involved in the metabolism of cellulose. Bevacezepine and miglitol, which are inhibitors of *α*-glucosidase, are commonly used drugs for lowering blood sugar levels and controlling type 2 diabetes. Zhang et al. demonstrated that Saskatoon berry leaf extract and subfractions potently suppressed mammalian *α*-glucosidase activity (EC 3.2.1.20), delayed or inhibited the absorption of carbohydrates, and significantly lowered postprandial blood glucose concentrations in a C57Bl6 mice model of high-fat diet-induced obesity and hyperglycemia [8]. In another study, Moghadasian et al. recently investigated the effects of adding 5% (*w*/*w*) SBP in the diet on mice body weight, glucose levels, cholesterol levels, triglyceride levels, and inflammatory factors during a 4-week study period. They found that the db/db mice (the experimental model for type 2 diabetes) treated with 5% SBP had a roughly 30% decrease in blood and urine glucose levels to those of db/db control mice. They also reported that the mice treated with SBP showed a different inflammatory marker profile between db/db and wild-type C57BL/6J groups: levels of leptin, TIMP-1 (tissue inhibitor of metalloproteinase), RANTES (regulated on activation and normal T cell expressed and secreted), VEGF (vascular endothelial growth factor), MCP-5 (monocyte chemoattractant protein-5), SCF (stem cell factor), and TARC (thymus- and activation-regulated chemokine) were higher in db/db mice than those in C57BL/6J mice. The mice treated with SBP also showed a lower level of IL-3 and sTNFRI (Soluble Tumor Necrosis Factor Receptor I) in C57BL/6J groups; SBP-treated db/db mice had lower lever of IL-3 and RANTES in db/db groups [34]. Thus, several studies have found Saskatoon berries to be able to interfere with the molecular pathways underlying diabetes.

Another area of research is dyslipoproteinemia, which is a classical risk factor for diabetes. Increased amounts of glyLDL or oLDL were frequently observed in diabetic and atherosclerotic cardiovascular disease patients. Illuminating the molecular mechanisms, previous studies demonstrated that glyLDL or oLDL stimulated the production of reactive oxygen species (ROS), induced the expression of heat shock factor-1 (HSF1), plasminogen activator inhibitor-1 (PAI-1), urokinase plasminogen activator (uPA), monocyte chemotactic protein-1 (MCP-1), intracellular adhesion molecule-1 (ICAM-1), and TNF*α*, as well as increasing the levels of NADPH oxidase (NOX) and reducing mitochondrial electron transport chain (mETC) enzyme activities in vascular endothelial cells (EC). Subsequent studies demonstrated that C3G or D3G, which is found in Saskatoon berries, prevented glyLDL or oLDL-induced oxidative stress, apoptosis, mitochondrial dysfunction, and impairment of cell viability in cultured vascular EC [26, 28, 35, 36]. In recent years, studies have explored the consequences of Saskatoon berry powder (SBP) on endoplasmic reticulum stress, relevant inflammatory, monocyte adhesion to the vascular wall, and fibrinolytic regulators in leptin receptor-knockout (db/db) diabetic mice. Studies have also investigated the significance of SBP on insulin resistance, as well as its influence on intestinal microbiota in high-fat/high-sucrose diet-induced obese mice [22, 33]. One recent study fed db/db mice with food containing 0.2%, 1%, 5%, and 20% SBP. After five weeks, they found that blood glucose levels decreased by 21%, 29%, 41%, and 17%, respectively, compared with the control group not fed SBP. Among the results, the 5% SBP group had the most significant blood sugar level drop. The db/db mice were fed with 5% of SBP for 4 weeks, and the study found that SBP powerfully inhibited monocyte adhesion to the aorta in diabetic db/db mice. SBP also effectively reduced the upregulation of fibrinolytic (such as PAI-1, uPA), stress (HSF1, NOX4), and inflammatory (MCP-1, ICAM-1, P-selectin, TNF*α*) regulators in the plasma, aorta or heart apex of db/db mice [27]. A related area of study between Saskatoon berries and diabetes is ER stress, which is correlated with insulin resistance, diabetes, obesity, and atherosclerosis. A common method for assessing misfolded or unfolded proteins during ER stress is the thioflavin T (ThT) assay. Studies demonstrated that ER stress was induced in the heart and aorta of db/db mice. After feeding the mice with 5% SBP for 4 weeks, the study found that SBP significantly inhibited the increase of ThT fluorescence and the UPR markers (GRP78/94, XBP-1, and CHOP) in the cardiovascular tissue of db/db mice [26]. In a recently published study, 24 Male C57BL/6J mice were split into three groups according to their diet and fed for 15 weeks: control, high-fat/high-sucrose (HF+HS), or HF+HS+5% SBP (HF+HS+B). The group with the HF+HS diet had increased plasma levels of glucose, insulin, homeostatic model assessment-insulin resistance (HOMA-IR), body weight, food intake, cholesterol, triglyceride, monocyte adhesion to vascular wall, TNF*α*, PAI-1, MCP-1, ICAM-1, uPA, and its receptor (uPAR) compared to the control group. The HF+HS+B diet group had a delayed increase in body weight and had suppressed HF+HS diet-induced disorders in regards to metabolic, fibrinolytic, and inflammatory changes.

## 5. Summary

Saskatoon berry is a native species of the North American plains. In the last two decades, the cultivation of SB has expanded from North America to various countries in Asia and Europe, including Finland, Poland, and Czech Republic. The major functional components of SB are polyphenolic compounds, primarily flavonoids. The seven subclasses of flavonoids include flavanes, flavanols (proanthocyanidins), flavanones, flavones, isoflavones, flavonols, and anthocyanidins. Mature SB contain significant levels of the anthocyanin, flavonol, and proanthocyanidin classes of flavonoids. The total anthocyanin concentration in SB is comparable to that of wild blueberry and higher than that in other small-fruited species such as raspberry, sea buckthorn, chokeberry, and strawberry. The major anthocyanins in SB are cyanidin-3-glucoside (C3G), cyanidin-3-galactoside (C3Ga), and delphinidin-3-glucoside (D3G). Others include cyanidin 3-O-arabinoside and cyanidin 3-O-xyloside. SB has been demonstrated to have many beneficial functions, including being an antioxidant and antiradical, as well as being potentially anticarcinogenic, anti-inflammatory, antidiabetic, vessel-protective, and neuroprotective. SB has potential preventative and therapeutic effects on diseases such as diabetes, cancers, inflammatory and cardiovascular diseases, obesity, neurodegenerative pathologies, and muscular degeneration.

Although SB were used in the treatment and prevention of diabetes by the Blackfoot Tribes of the North American Indigenous peoples, only recent scientific studies have explored its medical properties in controlled experiments. Some basic research has clarified certain functions. The nonpolar fraction of SB strongly inhibited aldose reductase, which is closely related to diabetic complications such as cataract, neuropathy, kidney disease, retinopathy, and atherosclerosis. The polar fraction of SB (mainly including phenolic acids, anthocyanins, and proanthocyanidins) lowered blood sugar levels. Saskatoon berry leaf extract and subfractions potently suppressed mammalian *α*-glucosidase activity and delayed the absorption of carbohydrates, significantly lowering postprandial blood glucose concentrations in a C57Bl6 mice model of high-fat diet-induced obesity and hyperglycemia. Studies have also demonstrated that mice fed a diet of 5% SBP had an obvious blood sugar drop and inhibited ER stress of cardiovascular tissue in diabetic db/db mice. Moreover, compared to mice fed a HF+HS diet, mice fed a diet of HF+HS+S (5% SBP) had lower plasma glucose, insulin, HOMA-IR, body weight, food intake, cholesterol, triglyceride, monocyte adhesion, TNF*α*, PAI-1, MCP-1, ICAM-1, and uPAR. The HF+HS+B diet significantly decreased the Firmicutes/Bacteroidetes ratio compared to the HF+HS diet on intestinal microbiota. With further validation, these studies suggest that Saskatoon berries may offer multiple new therapeutic approaches to targeting the molecular mechanisms underlying diabetic symptoms.

At present, research on Saskatoon berries and diabetes is still underdeveloped. There is a lack of long-term, large-population, randomized, clinical data with placebo-controlled and side-effect records. A variety of products that are easily accessible to consumers on the market also needs to be developed. However, Saskatoon berries, as natural food, may be one of the best alternatives for the prevention and treatment of diabetes in the future.

## 6. Limitations and Future Direction

The researches on the health benefits of Saskatoon berry is mainly in animal models. Human studies will be required to verify the beneficial effects of Saskatoon berry or its products in human healthy subjects and patients. The parts (fruits, peels, and leaves), extractions, or active compounds of Saskatoon berry responsible to its health benefits remains to be determined. Saskatoon berry is a seasonal fruit. The storage condition or a preferable format of Saskatoon berry should be optimized for using this functional food as health supplementation for chronic diseases. Future directions of the studies regarding the health benefits of Saskatoon berry are expected to emphasize the determination of the effects, safety, and proper dosages of Saskatoon berry products on common chronic diseases and identification of the active compounds in Saskatoon berry for its beneficial effect.
